# Supplementary material for: Quantitative Proteomics Reveals Fh15 as an Antagonist of TLR4 Downregulating the Activation of NF-κB, Inducible Nitric Oxide, Phagosome Signaling Pathways, and Oxidative Stress of LPS-Stimulated Macrophages
Source: Int J Mol Sci. 2025 Jul 18;26(14):6914. doi: 10.3390/ijms26146914 (PMC12294962; doi:10.3390/ijms26146914)
Supplement: Supplementary file 1 [file ijms-26-06914-s001.zip › ijms-3739501-supplementary.pdf]

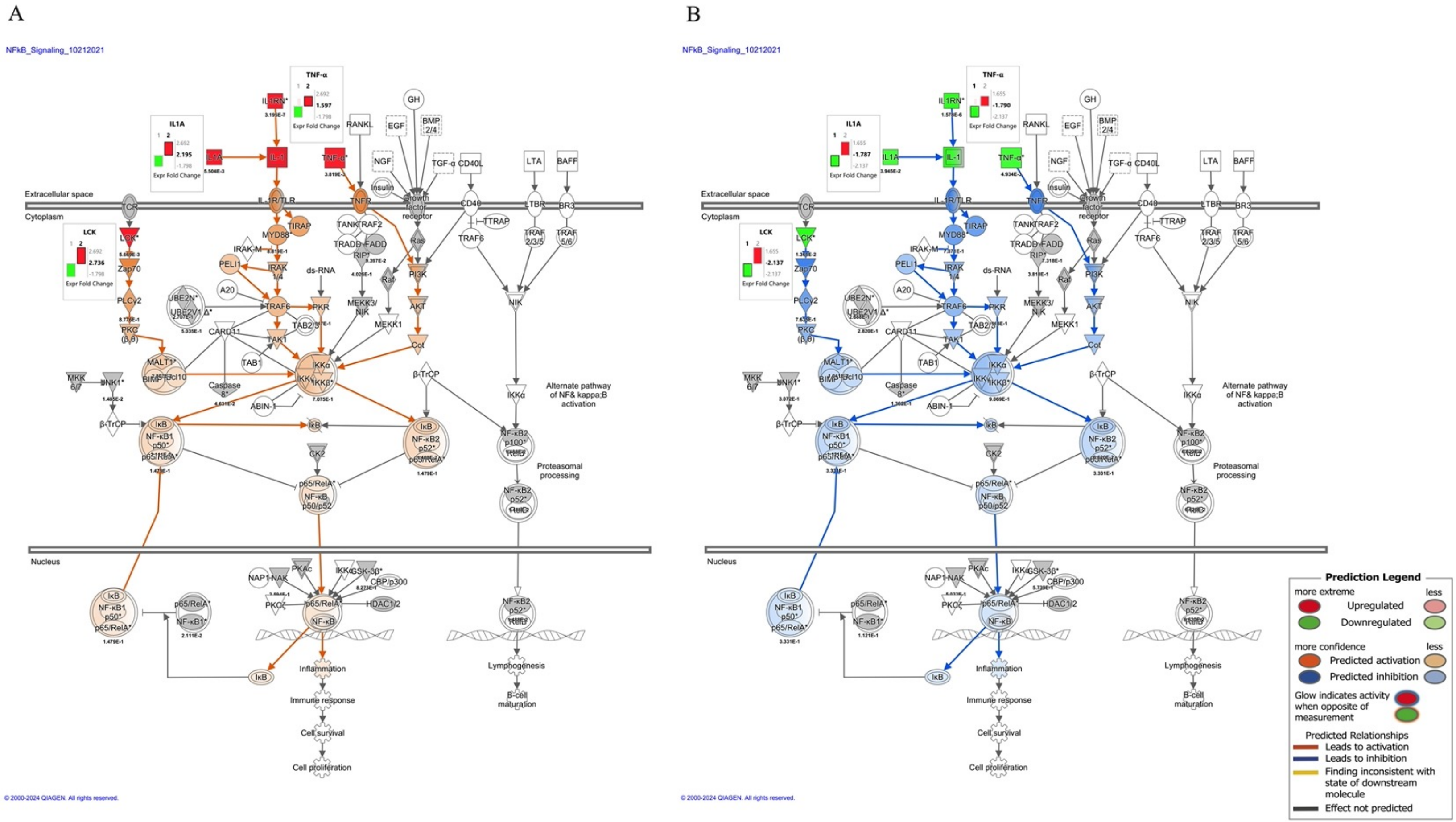

Figure S1. Proteins identified by IP-analysis associated to the NF-κB pathway. **(A)** Four proteins (IL1RN, Lck, IL1α, and TNF-α) were found 1.60 to 6.18-fold more upregulated in cells treated with LPS than in to PBS-control cells. **(B)** In contrast, these four proteins were found -4.47 to -1.1.80-fold downregulated in cells treated with Fh15 compared to the LPS.

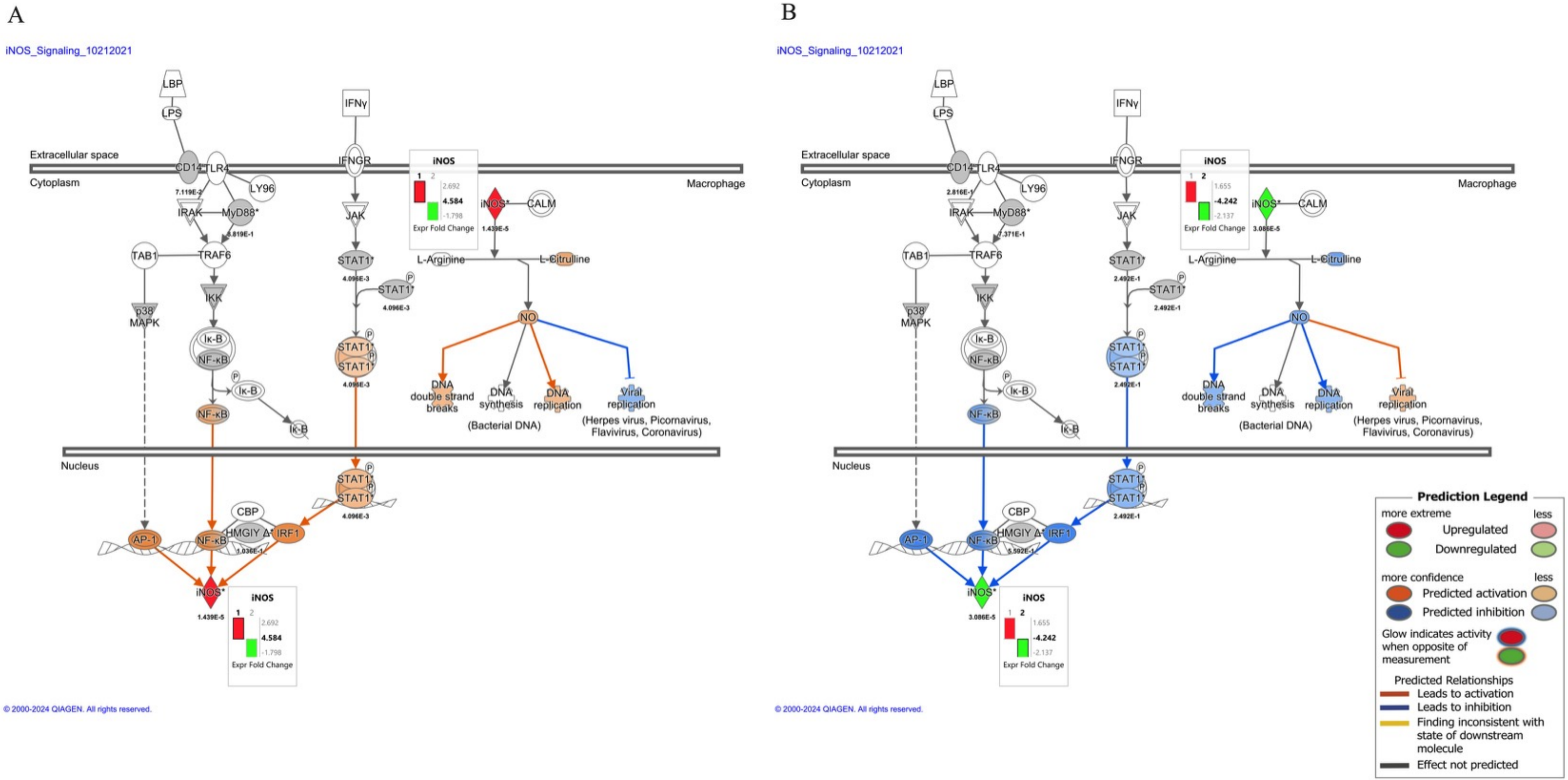

Figure S2. Proteins identified by Ingenuity Pathway Analysis (IPA) associated to the iNOS pathway. **(A)** iNOS was found 4.48-fold more upregulated in cells treated with LPS compared to the PBS-control. **(B)** In contrast, iNOS was found -4.24-fold less downregulated in cells treated with Fh15 compared to the LPS-control.

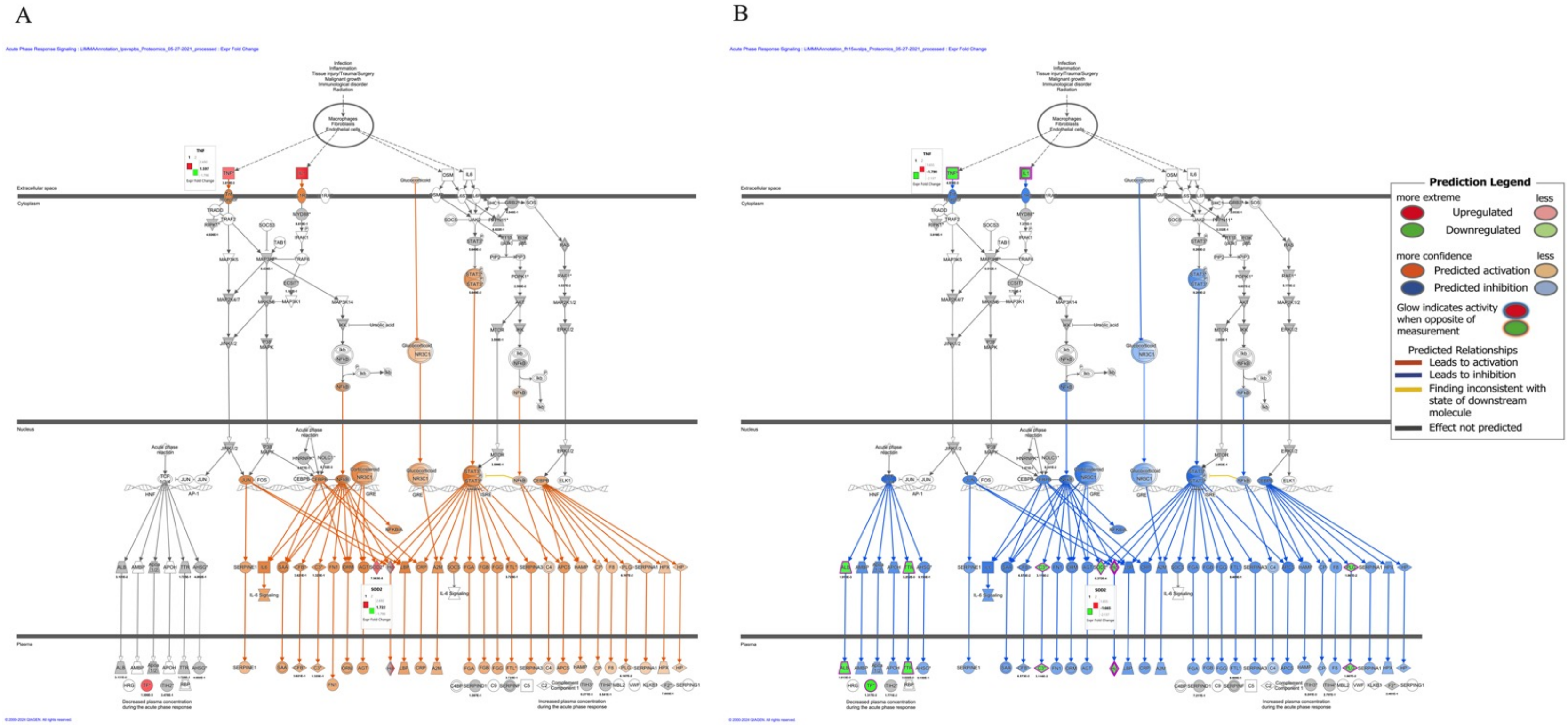

Figure S3. Proteins identified by Ingenuity Pathway Analysis (IPA) associated to the acute phase response signaling pathway. **(A)** SOD2 was 1.72-fold more upregulated in cells treated with LPS compared to the PBS-control. **(B)** In contrast, SOD2 was found -1.67-fold less downregulated in cells treated with Fh15 compared to the LPS-control.

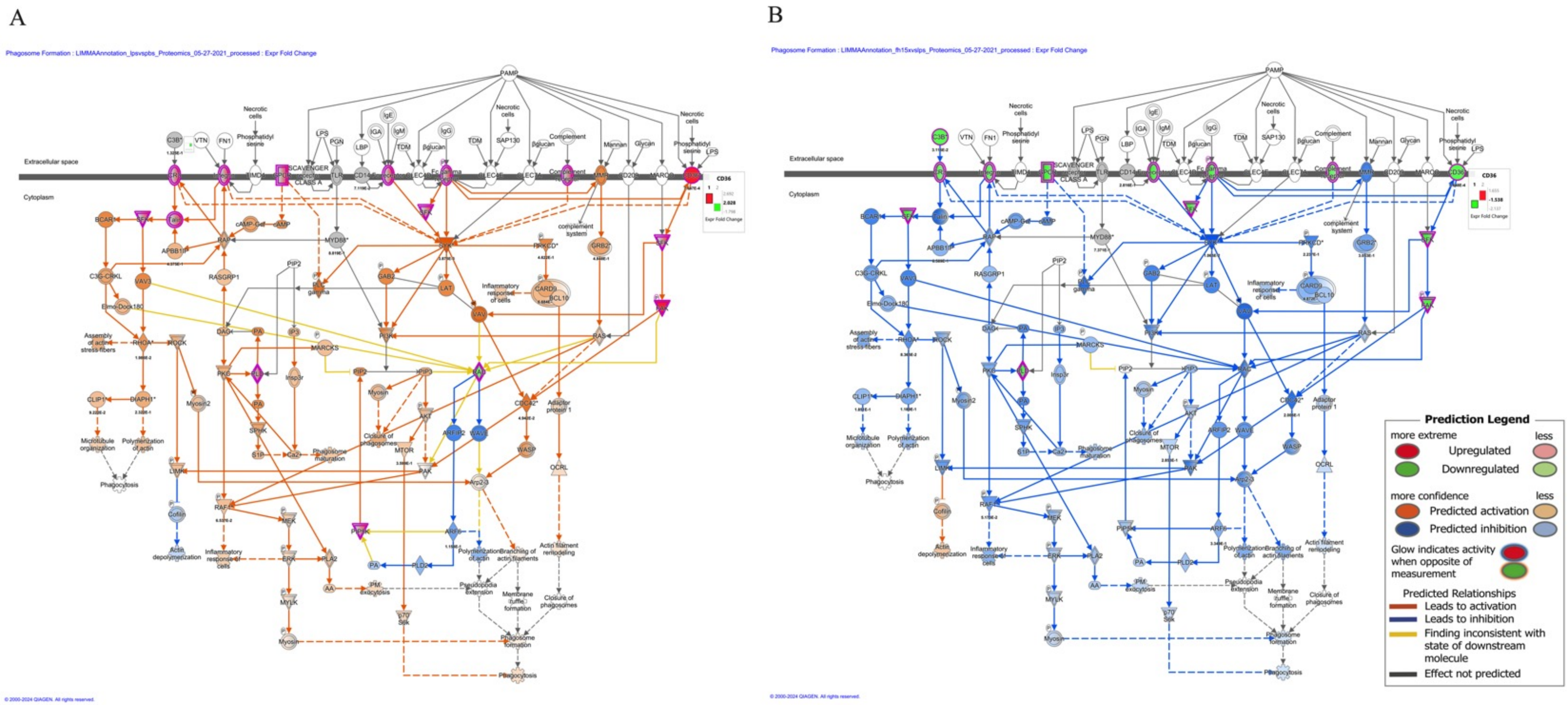

Figure S4. Proteins identified by Ingenuity Pathway Analysis (IPA) associated to the phagosome formation signaling pathway. **(A)** CD36 was 2.03-fold more upregulated in cells treated with LPS compared to the PBS-control. **(B)** In contrast, CD36 was found -1.54-fold less downregulated in cells treated with Fh15 compared to the LPS-control.

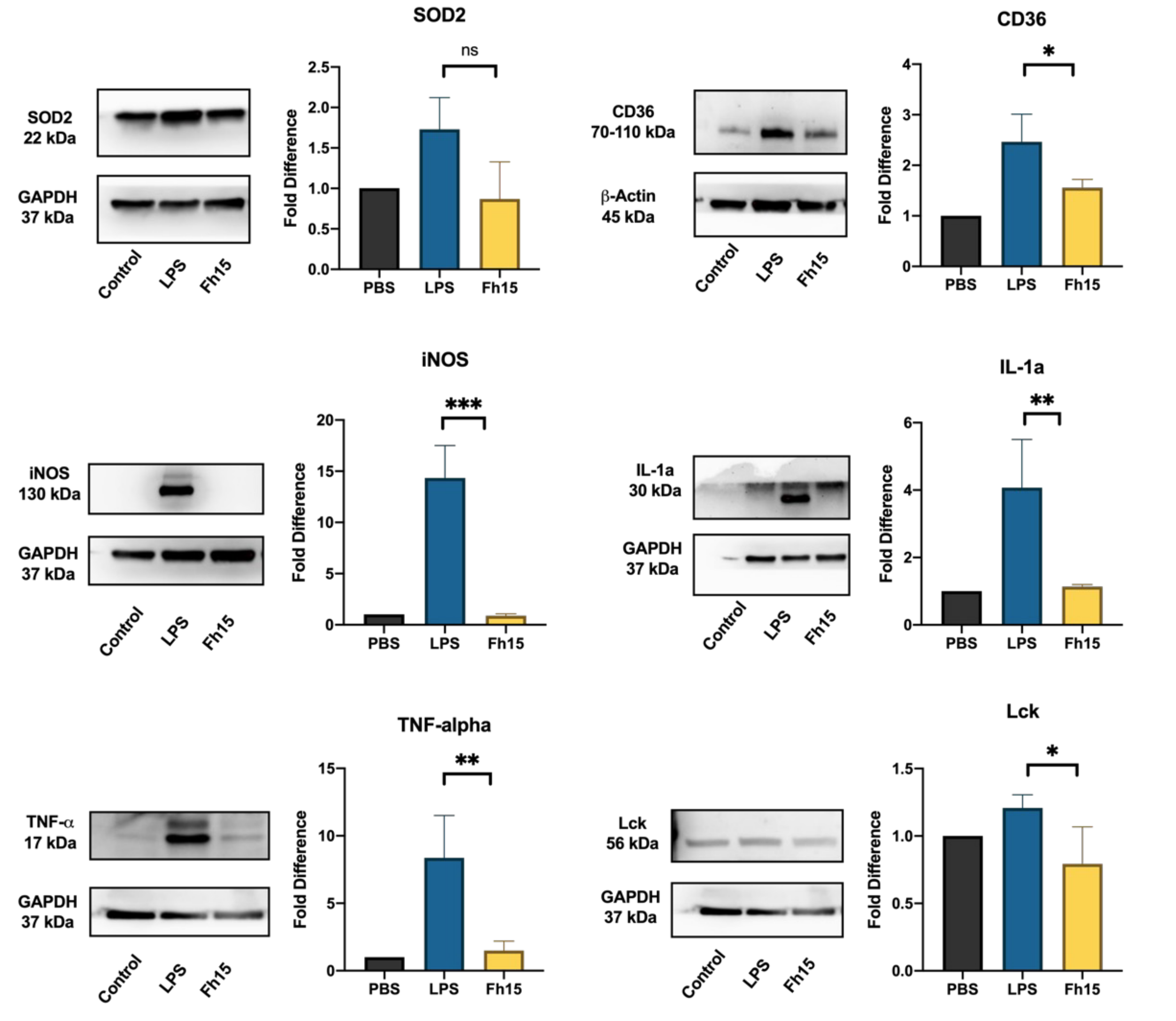

Figure S5. Fh15 suppresses the expression of proteins associated to several inflammatory pathways within macrophages-like cells. Fh15 significantly suppresses the expression of IL-1α (**p=0.0092), TNF-α (**p=0.0085), iNOS (***p<0.005), Lck (*p=0.0478), and CD36 (*p=0.0354). Fh15 also suppressed the expression of SOD2 although these effects were no statistically significant (ns).

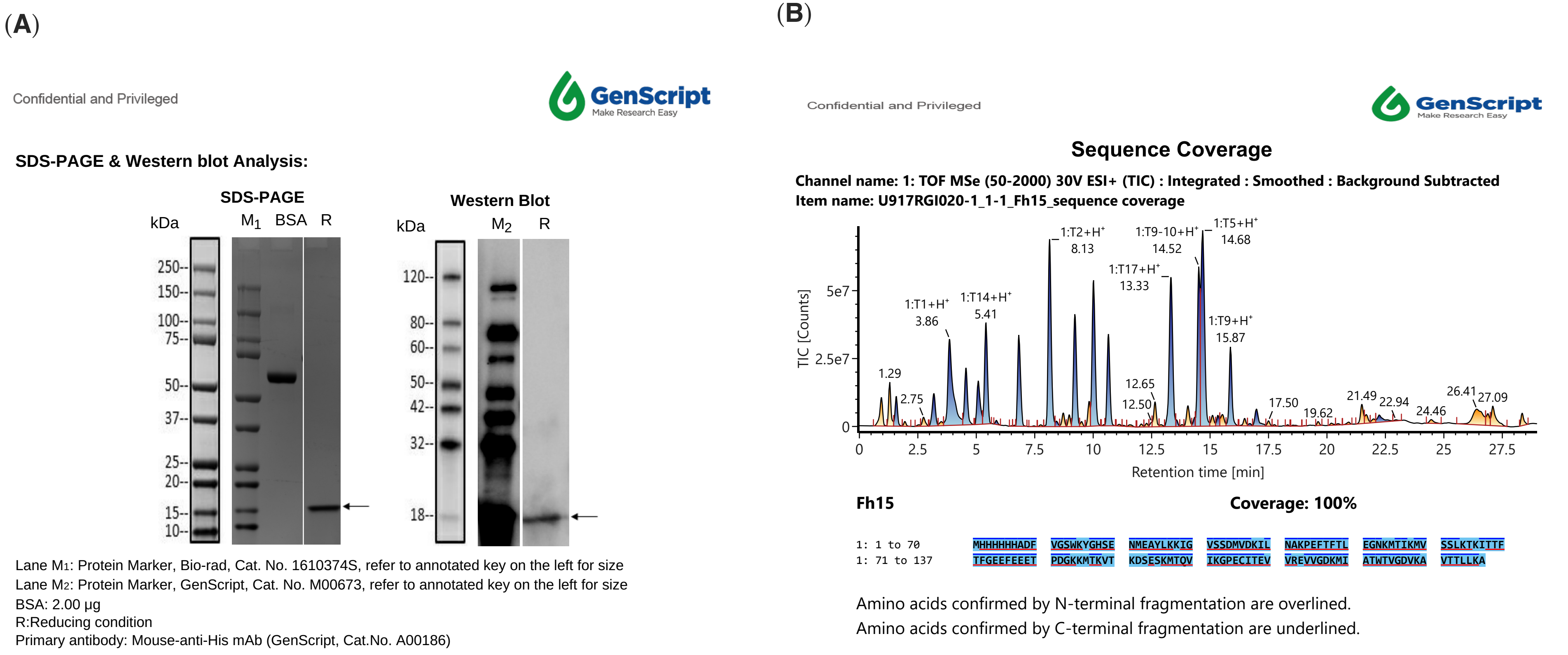

Figure S6. Western blot and Mass spectrometry analysis of purified Fh15. cDNA encoding amino acid sequence of Fh15 (GenBank ID M95291.1) was expressed in *Bacillus subtilis* as a fusion protein with a 6His at the amino terminus. After purification by a Ni+-agarose column and endotoxins removal purified Fh15 was concentrated by AMICON Ultra Centrifugal Filters (YM-3) and its concentration adjusted to 2.29 mg/mL. **(A)** Western blot using a mouse anti-Histidine tag monoclonal Antibody (Genscript Cat. No. A00186) was used to confirm purity of the purified protein. **(B)** The purity of Fh15 was >90% was confirmed by LC-MS/MS. The cDNA synthesis, cloning, expression and purification was performed in collaboration with GenScript, USA (Order # U917RGI020).

Table S1. List of 114 proteins identified and quantified by TMT-analysis, which were found upregulated by LPS and downregulated by Fh15 in RAW264.7 cells.

					Fh15 vs LPS		*LPS vs PBS*	
Symbol	**Gene Name**	**ID**	**Location**	**Type(s)**	**Fold Change**	**p-value**	**Fold Change**	**p-value**
IL1RN	interleukin 1 receptor antagonist	Q3TBV5	Extracellular Space	cytokine	-4.469	0.00000158	6.181	3.19E-07
PTGS2	prostaglandin-endoperoxide synthase 2	Q05769	Cytoplasm	enzyme	-4.764	0.0000339	5.551	0.00000979
SERPINB2	serpin family B member 2	F6ZFV0	Extracellular Space	other	-4.898	0.00000349	5.426	0.00000113
UPP1	uridine phosphorylase 1	Q5SUC6	Cytoplasm	enzyme	-5.305	0.00000517	5.255	0.00000456
NOS2	nitric oxide synthase 2	P29477	Cytoplasm	enzyme	-4.242	0.0000309	4.584	0.0000144
ACOD1	aconitate decarboxylase 1	P54987	Cytoplasm	enzyme	-3.856	0.00000702	4.208	0.00000267
Ifit1	interferon-induced protein with tetratricopeptide repeats 1	Q64282	Cytoplasm	other	-3.473	0.00000657	3.931	0.0000024
NOS3	nitric oxide synthase 3	P70313	Cytoplasm	enzyme	-3.771	0.000114	3.667	0.000116
SLC7A11	solute carrier family 7 member 11	Q9WTR6	Plasma Membrane	transporter	-3.107	0.00164	3.564	0.00249
ODC1	ornithine decarboxylase 1	A0A1Y7VJC5	Cytoplasm	enzyme	-3	0.0000198	3.417	0.0000149
Csta1 (includes others)	cystatin A1	B2RV77	Cytoplasm	other	-3.354	0.000356	3.231	0.000339
SLC15A3	solute carrier family 15 member 3	Q8BPX9	Cytoplasm	transporter	-2.968	0.000491	3.205	0.0000155
IFIT2	interferon induced protein with tetratricopeptide repeats 2	Q6GTM0	Cytoplasm	other	-2.213	0.0024	2.992	0.0000976
HMOX1	heme oxygenase 1	P14901	Cytoplasm	enzyme	-2.507	0.0000157	2.871	0.00000692
FABP4	fatty acid binding protein 4	P04117	Cytoplasm	transporter	-2.055	0.00534	2.824	0.00116
Ifit3/Ifit3b	interferon-induced protein with tetratricopeptide repeats 3	Q64345	Cytoplasm	other	-2.472	0.00157	2.765	0.000443
LCK	LCK proto-oncogene, Src family tyrosine kinase	P06240	Cytoplasm	kinase	-2.137	0.0137	2.736	0.00567
PLEK	pleckstrin	Q5F270	Cytoplasm	other	-2.037	0.0000619	2.713	0.00000779
FYN	FYN proto-oncogene, Src family tyrosine kinase	P39688-2	Plasma Membrane	kinase	-1.608	0.0126	2.692	0.00137
ISG15	ISG15 ubiquitin like modifier	Q64339	Cytoplasm	other	-1.837	0.000693	2.651	0.0000201
KCTD8	potassium channel tetramerization domain containing 8	Q50H33-2	Other	other	-2.74	0.00232	2.637	0.00332
SRC	SRC proto-oncogene, non-receptor tyrosine kinase	P05480	Cytoplasm	kinase	-1.643	0.00701	2.633	0.000955
YES1	YES proto-oncogene 1, Src family tyrosine kinase	Q04736	Cytoplasm	kinase	-1.643	0.00701	2.633	0.000955
SRGN	serglycin	P13609	Cytoplasm	other	-1.976	0.00147	2.63	0.00828

SQSTM1	sequestosome 1	Q64337-2	Cytoplasm	transcription regulator	-2.25	0.0000825	2.601	0.0000327
GPR84	G protein-coupled receptor 84	Q8CIM5	Plasma Membrane	G-protein coupled receptor	-1.97	0.0286	2.589	0.000721

					Fh15 vs LPS		***LPS vs PBS***	
Symbol	**Gene Name**	**ID**	**Location**	**Type(s)**	**Fold Change**	**p-value**	**Fold Change**	**p-value**
ANXA7	annexin A7	A0A286YCW4	Plasma Membrane	ion channel	-1.935	0.025	2.5	0.00614
Kctd12b	potassium channel tetramerisation domain containing 12b	Q8C7J6	Plasma Membrane	ion channel	-2.418	0.0104	2.471	0.00405
Ccl9	C-C motif chemokine ligand 9	P51670	Extracellular Space	cytokine	-1.806	0.00413	2.362	0.000211
SPP1	secreted phosphoprotein 1	D3Z4N2	Extracellular Space	cytokine	-1.519	0.0238	2.334	0.00341
SLC2A1	solute carrier family 2 member 1	P17809	Plasma Membrane	transporter	-1.892	0.00121	2.32	0.000111
Oasl1	2'-5' oligoadenylate synthetase-like 1	Q8VI94	Other	other	-2.03	0.000282	2.304	0.0000673
KCTD12	potassium channel tetramerization domain containing 12	Q6WVG3	Plasma Membrane	ion channel	-1.895	0.00296	2.275	0.00226
Lilrb4a/Lilrb4b	leukocyte immunoglobulin-like receptor, subfamily B, member 4A	Q64281-2	Plasma Membrane	other	-1.765	0.00184	2.231	0.000241
LTF	lactotransferrin	P08071	Extracellular Space	other	-1.999	0.00811	2.225	0.0107
IL1A	interleukin 1 alpha	P01582	Extracellular Space	cytokine	-1.787	0.0394	2.195	0.0055
CDS2	CDP-diacylglycerol synthase 2	F6S4G2	Cytoplasm	enzyme	-1.687	0.0462	2.109	0.000891
PFKFB3	6-phosphofructo-2-kinase/fructose-2,6-biphosphatase 3	A0A0A6YY64	Cytoplasm	enzyme	-1.956	0.00107	2.089	0.000384
GCH1	GTP cyclohydrolase 1	Q05915	Cytoplasm	enzyme	-1.648	0.000507	2.085	0.000119
HCK	HCK proto-oncogene, Src family tyrosine kinase	P08103-2	Cytoplasm	kinase	-1.529	0.00506	2.08	0.000311
Fcgr2b	Fc receptor, IgG, low affinity IIb	P08101	Plasma Membrane	transmembrane receptor	-1.922	0.000401	2.062	0.000127
CNDP2	carnosine dipeptidase 2	A0A494B9U6	Cytoplasm	peptidase	-1.76	0.0494	2.06	0.0102
CMPK2	cytidine/uridine monophosphate kinase 2	Q3U5Q7	Cytoplasm	kinase	-1.81	0.00212	2.035	0.000552
CD36	CD36 molecule (CD36 blood group)	A0A0G2JFB7	Plasma Membrane	transmembrane receptor	-1.538	0.000565	2.028	0.000765
Phf11d (includes others)	PHD finger protein 11D	A6H5X4-3	Nucleus	other	-1.675	0.000656	1.985	0.000276
LMBRD1	LMBR1 domain containing 1	Q8K0B2-3	Cytoplasm	transporter	-1.556	0.0139	1.981	0.00805
TMEM120A	transmembrane protein 120A	Q8C1E7	Plasma Membrane	ion channel	-1.71	0.0192	1.973	0.0127
Fcgr3	Fc receptor, IgG, low affinity III	P08508	Plasma Membrane	transmembrane receptor	-1.823	0.000109	1.971	0.000067

ELL2	elongation factor for RNA polymerase II 2	A0A1Y7VMV7	Nucleus	transcription regulator	-1.793	0.00167	1.961	0.000285
FNDC3A	fibronectin type III domain containing 3A	E0CXY0	Cytoplasm	other	-1.79	0.00548	1.948	0.00472
PLAUR	plasminogen activator, urokinase receptor	P35456	Plasma Membrane	transmembrane receptor	-1.521	0.0319	1.932	0.000813

					Fh15 vs LPS		***LPS vs PBS***	
Symbol	**Gene Name**	**ID**	**Location**	**Type(s)**	**Fold Change**	**p-value**	**Fold Change**	**p-value**
ADAM8	ADAM metallopeptidase domain 8	E9Q359	Plasma Membrane	peptidase	-1.607	0.0298	1.915	0.000967
GK2	glycerol kinase 2	Q9WU65	Cytoplasm	kinase	-1.685	0.0000791	1.913	0.0000343
EPB41L2	erythrocyte membrane protein band 4.1 like 2	A0A1W2P7I2	Plasma Membrane	other	-1.889	0.0388	1.898	0.0408
SGPP1	sphingosine-1-phosphate phosphatase 1	Q9JI99	Cytoplasm	phosphatase	-1.587	0.0412	1.871	0.0347
CUL4A	cullin 4A	Q3TCH7	Nucleus	enzyme	-1.709	0.0494	1.866	0.00559
CPD	carboxypeptidase D	O89001	Extracellular Space	peptidase	-1.593	0.000445	1.861	0.000342
TF	transferrin	F7BAE9	Extracellular Space	transporter	-2.084	0.00132	1.854	0.00131
HELZ2	helicase with zinc finger 2	E9QAM5	Nucleus	transcription regulator	-1.596	0.000316	1.853	0.000114
Mpeg1	macrophage expressed gene 1	E9QN37	Cytoplasm	transporter	-1.774	0.000976	1.842	0.000259
SCIMP	SLP adaptor and CSK interacting membrane protein	Q3UU41	Plasma Membrane	other	-1.51	0.0354	1.823	0.0151
Aldh3b3	aldehyde dehydrogenase 3 family, member B3	F6QPV9	Cytoplasm	enzyme	-1.64	0.0418	1.808	0.0215
EEA1	early endosome antigen 1	A0A1W2P7A6	Cytoplasm	other	-1.619	0.0432	1.773	0.00501
PTPRJ	protein tyrosine phosphatase receptor type J	Q64455	Plasma Membrane	phosphatase	-1.639	0.0186	1.727	0.0127
FOSL2	FOS like 2, AP-1 transcription factor subunit	P47930-2	Nucleus	transcription regulator	-1.567	0.00338	1.726	0.0227
GNA13	G protein subunit alpha 13	P27601	Plasma Membrane	enzyme	-1.696	0.0154	1.724	0.0402
SOD2	superoxide dismutase 2	P09671	Cytoplasm	enzyme	-1.665	0.000527	1.722	0.0000756
PIK3AP1	phosphoinositide-3-kinase adaptor protein 1	Q9EQ32-3	Cytoplasm	other	-1.623	0.000808	1.686	0.000185
LRRFIP1	LRR binding FLII interacting protein 1	A0A087WPT0	Cytoplasm	transcription regulator	-1.546	0.0121	1.658	0.00677
PTK2B	protein tyrosine kinase 2 beta	Q9QVP9	Cytoplasm	kinase	-1.83	0.00409	1.655	0.0111
RHOT2	ras homolog family member T2	Q8JZN7	Cytoplasm	enzyme	-1.542	0.0193	1.655	0.0183
SYNJ1	synaptojanin 1	F7CD11	Cytoplasm	phosphatase	-1.559	0.035	1.63	0.0441
LASP1	LIM and SH3 protein 1	A2A6H1	Cytoplasm	transporter	-1.598	0.0179	1.626	0.0084
COPS7A	COP9 signalosome subunit 7A	D3Z0S0	Cytoplasm	other	-1.651	0.0433	1.622	0.0129

JUNB	JunB proto-oncogene, AP-1 transcription factor subunit	P09450	Nucleus	transcription regulator	-1.633	0.0000777	1.612	0.00119
TNF	tumor necrosis factor	P06804	Extracellular Space	cytokine	-1.79	0.00493	1.597	0.00382
ERMP1	endoplasmic reticulum metallopeptidase 1	Q3UVK0-2	Cytoplasm	other	-1.527	0.0118	1.58	0.00581

					Fh15 vs LPS		***LPS vs PBS***	
Symbol	**Gene Name**	**ID**	**Location**	**Type(s)**	**Fold Change**	**p-value**	**Fold Change**	**p-value**
NBEA	neurobeachin	Q9EPN1-4	Cytoplasm	other	-1.654	0.0394	1.511	0.025
FBLN1	fibulin 1	Q08879	Extracellular Space	other	-1.739	0.00242	1.503	0.00527
ILK	integrin linked kinase	A0A1B0GRF6	Plasma Membrane	kinase	-1.505	0.0054	1.502	0.00283
NT5DC1	5'-nucleotidase domain containing 1	Q8C5P5-2	Other	other	1.621	0.000363	-1.515	0.000281
MBNL3	muscleblind like splicing regulator 3	S4R267	Nucleus	other	1.626	0.00869	-1.516	0.0043
FAU	FAU ubiquitin like and ribosomal protein S30 fusion	Q642K5	Cytoplasm	other	1.563	0.00276	-1.536	0.0018
UGGT2	UDP-glucose glycoprotein glucosyltransferase 2	G3UXP5	Cytoplasm	enzyme	1.609	0.0144	-1.541	0.0287
RPL29	ribosomal protein L29	A0A1L1STJ3	Cytoplasm	other	1.575	0.0112	-1.573	0.00223
GSS	glutathione synthetase	H3BKH4	Cytoplasm	enzyme	1.676	0.0415	-1.583	0.0125
FADS2	fatty acid desaturase 2	Q9Z0R9	Plasma Membrane	enzyme	1.937	0.000441	-1.613	0.000462
YY1	YY1 transcription factor	Q00899	Nucleus	transcription regulator	1.572	0.00214	-1.619	0.000459
YY2	YY2 transcription factor	Q3TTC2	Nucleus	transcription regulator	1.572	0.00214	-1.619	0.000459
DTYMK	deoxythymidylate kinase	D3Z3R3	Cytoplasm	kinase	1.578	0.000892	-1.627	0.000498
MBNL1	muscleblind like splicing regulator 1	A0A0A6YXL7	Nucleus	other	1.803	0.0158	-1.64	0.00241
HNRNPLL	heterogeneous nuclear ribonucleoprotein L like	Q921F4-5	Plasma Membrane	other	1.646	0.000759	-1.642	0.0000609
LYAR	Ly1 antibody reactive	D3YU83	Plasma Membrane	other	1.527	0.0146	-1.647	0.0016
NDUFA4	NDUFA4 mitochondrial complex associated	Q62425	Cytoplasm	enzyme	1.502	0.00305	-1.683	0.00116
CSNK1A1	casein kinase 1 alpha 1	F6YBC9	Cytoplasm	kinase	1.666	0.00302	-1.689	0.000164
TOP2A	DNA topoisomerase II alpha	Q01320	Nucleus	enzyme	1.751	0.000278	-1.693	0.000355
DEPDC1B	DEP domain containing 1B	Q8BH88	Cytoplasm	other	1.944	0.0019	-1.703	0.000781
SDHC	succinate dehydrogenase complex subunit C	Q9CZB0	Cytoplasm	enzyme	1.619	0.000536	-1.712	0.0000652
ADSL	adenylosuccinate lyase	A0A0G2JFI8	Cytoplasm	enzyme	1.549	0.00261	-1.731	0.000598
TSFM	Ts translation elongation factor, mitochondrial	D3Z4M7	Cytoplasm	translation regulator	1.603	0.0114	-1.745	0.000884
PRC1	protein regulator of cytokinesis 1	Q99K43	Nucleus	other	1.711	0.0000846	-1.746	0.000363

					Fh15 vs LPS		*LPS vs PBS*	
Symbol	**Gene Name**	**ID**	**Location**	**Type(s)**	**Fold Change**	**p-value**	**Fold Change**	**p-value**
RBM27	RNA binding motif protein 27	Q5SFM8-2	Nucleus	other	1.584	0.0275	-1.808	0.00668
PLOD1	procollagen-lysine,2-oxoglutarate 5-dioxygenase 1	A8Y5E6	Cytoplasm	enzyme	1.782	0.0000561	-1.812	0.000147
GNL2	G protein nucleolar 2	B1ASC2	Nucleus	enzyme	1.722	0.000331	-1.82	0.00221
UBAP2	ubiquitin associated protein 2	F6WTC8	Cytoplasm	other	1.771	0.00429	-1.851	0.000986
NDUFV1	NADH:ubiquinone oxidoreductase core subunit V1	A0A494BA02	Cytoplasm	enzyme	1.581	0.000689	-1.859	0.000217
CEP55	centrosomal protein 55	Q8BT07-2	Cytoplasm	other	1.744	0.000274	-1.946	0.000951
NDUFV2	NADH:ubiquinone oxidoreductase core subunit V2	Q9D6J6-2	Cytoplasm	enzyme	2.012	0.0145	-2.216	0.0049
KPNA2	karyopherin subunit alpha 2	F2Z431	Nucleus	other	2.52	0.00156	-2.328	0.000766
NDUFA6	NADH:ubiquinone oxidoreductase subunit A6	A0A2R8VI90	Cytoplasm	enzyme	2.396	0.00236	-2.556	0.000539
